# From Cannabinoids and Neurosteroids to Statins and the Ketogenic Diet: New Therapeutic Avenues in Rett Syndrome?

**DOI:** 10.3389/fnins.2019.00680

**Published:** 2019-07-02

**Authors:** Francisco Melo Mouro, Catarina Miranda-Lourenço, Ana Maria Sebastião, Maria José Diógenes

**Affiliations:** ^1^Instituto de Farmacologia e Neurociências, Faculdade de Medicina, Universidade de Lisboa, Lisbon, Portugal; ^2^Instituto de Medicina Molecular João Lobo Antunes, Faculdade de Medicina, Universidade de Lisboa, Lisbon, Portugal

**Keywords:** Rett syndrome, GABA_A_R, epilepsy, cholesterol, ketogenic diet, cannabinoids, neurosteroids

## Abstract

Rett syndrome (RTT) is an X-linked neurodevelopmental disorder caused mainly by mutations in the *MECP2* gene, being one of the leading causes of mental disability in females. Mutations in the *MECP2* gene are responsible for 95% of the diagnosed RTT cases and the mechanisms through which these mutations relate with symptomatology are still elusive. Children with RTT present a period of apparent normal development followed by a rapid regression in speech and behavior and a progressive deterioration of motor abilities. Epilepsy is one of the most common symptoms in RTT, occurring in 60 to 80% of RTT cases, being associated with worsening of other symptoms. At this point, no cure for RTT is available and there is a pressing need for the discovery of new drug candidates to treat its severe symptoms. However, despite being a rare disease, in the last decade research in RTT has grown exponentially. New and exciting evidence has been gathered and the etiopathogenesis of this complex, severe and untreatable disease is slowly being unfolded. Advances in gene editing techniques have prompted cure-oriented research in RTT. Nonetheless, at this point, finding a cure is a distant reality, highlighting the importance of further investigating the basic pathological mechanisms of this disease. In this review, we focus our attention in some of the newest evidence on RTT clinical and preclinical research, evaluating their impact in RTT symptomatology control, and pinpointing possible directions for future research.

## Introduction

In 1966, the neuro pediatrician Andreas Rett described and published a report concerning a neurodevelopmental disorder affecting females (Rett, 1966; Percy, 2016). In this report, Dr. Rett described a disease characterized as having an early onset of developmental delay followed by a rapid regression, loss of communication ability and fine motor capabilities, as well as, the occurrence of stereotypic hand movements and periodic breathing during wakefulness (Percy, 2016). Today, this disease is known as Rett syndrome (RTT). RTT is a severe neurodevelopmental X-linked disorder affecting almost exclusively female patients, with prevalence of approximately 1:10000 live births (Segatto et al., 2014) and with a high rate of sporadic mutations (Patankar, 2014). Despite its rareness, RTT is still the second most common cause of severe mental retardation in females (De Felice et al., 2012). The majority of RTT cases are characterized by an archetypal clinical scenario, comprised by loss of acquired cognitive, social, and motor skills, in an usual four-stage neurologic regression, occurring simultaneously with the development of autistic behavior (Hagberg, 2002; Neul et al., 2010; De Felice et al., 2012; Braat and Kooy, 2015). According to Hagberg, stage I is named “early onset stagnation,” occurs 6 months to 1.5 years after birth and is characterized by delays on the developmental progress. Subsequently, stage II, or “developmental regression,” occurs from year 1 to 4, being characterized by rapid loss of acquired skills and communication and manifestation of mental deficiency. Stage III is termed “pseudostationary period,” being characterized by apparent preserved ambulatory ability and restitution of some communicative abilities, while a slow neuromotor regression occurs. Lastly, stage IV or “later motor deterioration,” onsets when the ambulation of stage III ceases and its characterized by complete wheelchair dependence, severe disability, wasting and distal distortion. RTT patients can also experience gastrointestinal problems, hypoplasia, early-onset osteoporosis, bruxism and screaming spells (Hagberg, 2002). Reductions in brain weight and cortical atrophy/microencephaly, resulting in a smaller head circumference, were early reported as a common feature occurring in children diagnosed with RTT (Jellinger et al., 1988). As thoroughly reviewed in Kyle et al. (2018), metabolic complications are also known as a common feature in RTT (Justice et al., 2013; Segatto et al., 2014). Also, as revised in Shulyakova et al. (2017), important anomalies in mitochondrial structure and function (altered electron transport chain complex function, increased oxidative stress and elevated levels of lactate and pyruvate in blood and cerebrospinal fluid) have been demonstrated in RTT. The clinical diagnosis of RTT is performed following a battery of co-existing and well-defined set of inclusion and exclusion criteria, which were recently revised (Neul et al., 2010; Kyle et al., 2018).

In Amir et al. (1999), using a systematic gene screening approach, identified mutations in the gene *MECP2* as cause of some cases of RTT. Today, several mutations in the X-linked *MECP2* gene are identified, being acknowledged as the cause of 95% of the classical RTT cases and of 40–50% of the atypical RTT cases (Neul et al., 2010; Zhang et al., 2017), resulting in a wide genetic and phenotypic heterogeneity of this disease (De Felice et al., 2012). *MECP2* is a globally expressed pleiotropic factor, assuming a key role in maintaining homeostasis in different cells and systems (Shulyakova et al., 2017). As expected for a neurological condition (Neul and Zoghbi, 2004), in RTT the higher levels of *MECP2* in the body are expressed in the brain (Shahbazian, 2002). Confirming the importance of *MECP2* presence in the brain, the selective elimination of this gene from neurons (Guy et al., 2007) and oligodendrocytes (Nguyen et al., 2012) triggers RTT-like phenotype in mice (Shulyakova et al., 2017). In atypical RTT variants, mutations in other loci rather than the *MECP2* have been identified (Neul et al., 2010).

A diversity of distinctive variant forms of RTT has been proposed, each of which presents different clinical features. Some of these variants have been identified and described in relatively small populations, which leads to difficulties in the definition of clear clinical characteristics (Neul et al., 2010). Nevertheless, according to Neul et al. (2010), there are three distinctive variants of RTT that have been amply identified and that are well characterized: (1) the preserved speech variant (Zappella, 1992), (2) the congenital variant (Rolando, 1985), and (3) the early seizure variant (Hanefeld, 1985). From these three atypical forms of RTT, the preserved speech variant, or Zappella variant, is the most common one. This variant has clearly defined clinical features and mutations on the *MECP2* gene are identified in most of the diagnosed cases (Zappella, 1992; Renieri et al., 2009; Neul et al., 2010). On the contrary, in diagnosed cases belonging to the congenital and early seizure variants, mutations in the *MECP2* gene are rarely found, while mutations in different genes are reported (Huppke et al., 2000; Archer et al., 2006; Neul et al., 2010; De Felice et al., 2012). Namely, mutations in the *CDKL5* gene have been described in both males and females diagnosed with early-seizure-onset RTT variant (Mari et al., 2005; Scala et al., 2005; Zhao et al., 2014; Zhang et al., 2017). On the other hand, mutations in the FOXG1 gene have been identified in the congenital RTT variant (Zhang et al., 2017), first described by Rolando (1985). The congenital variant of RTT is clinically characterized by hypotonia and developmental delay occurring earlier than in classical RTT variant (Rolando, 1985; Jacob et al., 2009). Importantly, the majority of children diagnosed with the congenital variant of RTT do not present mutations in the *MECP2* or *CDKL5* mutations (Jacob et al., 2009).

The available treatment for RTT is mainly symptomatic. With appropriate social and familiar care, attention to orthopedic complications, physiotherapy to treat and ease muscle rigidity, control of epileptic episodes, and a balanced and healthy nutrition, women with RTT can survive until middle age and older age (Kyle et al., 2018). There is a sudden death rate of 26% in RTT and patients mainly perish due to cardiac complications, respiratory infection and respiratory failure (Kyle et al., 2018). Most of the preclinical and clinical studies in RTT aims at finding ways to prevent or control epileptic episodes, due to the importance that it has for the outcome of RTT prognosis (Krajnc, 2015; Clarke and Abdala Sheikh, 2018). Therefore, preclinical research in RTT is majorly focused on finding ways of correcting detrimental modifications in neurotransmission occurring in this disease, using *Mecp2*-null mouse models (Clarke and Abdala Sheikh, 2018). Recently, however, a paradigm shift has been proposed. Through gene therapy it was proven that it is possible to reverse RTT symptoms in diseased adult mice by re-activating *Mecp2* expression (Guy et al., 2007). Also, a few years later, it was shown that switching-off the production of Mecp2 protein in adult mice leads to the development of symptoms equivalent to those of mice born with the mutation (McGraw et al., 2011; Clarke and Abdala Sheikh, 2018). Considering that, by rule, neurodevelopmental disorders tend to be non-reversible, such results were received with considerable enthusiasm (Clarke and Abdala Sheikh, 2018). Thus, these studies showed that gene therapy would have to necessarily deliver a working *MECP2* gene throughout the patient life (it would not be sufficient to do it just on child development) and that treatment could be administered regardless of disease stage and/or age (Clarke and Abdala Sheikh, 2018). Although exciting, the above-mentioned discoveries have to be taken carefully. Indeed, in order to work, *MECP2* gene therapy would have to deliver the precise amount of MECP2 protein in each cell of the body, as too much or too few MECP2 protein can trigger RTT-like symptoms (Clarke and Abdala Sheikh, 2018). As a particularly harsh example, in *MECP2* duplication syndrome, an overproduction of MECP2 protein leads to mental disability and autistic-like behavior (Moretti and Zoghbi, 2006; Ramocki et al., 2009). Moreover, even small deviations from the necessary MECP2 protein levels are related with modifications in brain function, which can lead to cognitive and mental disability (Chao and Zoghbi, 2012). Therefore, as mentioned in Clarke and Abdala Sheikh (2018), gene therapy in RTT delivers two major challenges: (1) it potentially means that it would be necessary to deliver the exact right amount of *MECP2* to every cell and (2) in females, it would be necessary to avoid delivering additional copies of the gene to the cells that already express a healthy copy; two pitfalls that are extremely difficult to overcome with the available technology.

At this point, although possible, finding a cure for RTT is a distant reality. In the meanwhile, it is more important than ever to find new treatments to alleviate symptoms, reduce pain and discomfort and increase the quality of life of both the patient and the caregiver. Therefore, in this review we will turn our attention to new discoveries which show potential in RTT preclinical investigation.

## Dysfunctional GABAR Signaling in RTT: Implications for Symptomatology

Rett syndrome is characterized by structural and molecular deficiencies in synaptic transmission. Evidence displays important modifications in basal transmission, in short and long-term plasticity processes and in neurotransmitter release (Boggio et al., 2010). Studies using different *Mecp2* genetic mutated mice have made it possible to understand that individual phenotypic characteristics of RTT can be associated with dysfunctions in very specific neuronal populations. Indeed, the selective deletion of the *Mecp2* gene from different neuronal populations, in different ages and in distinctive brain regions accounts for specific phenotypical traits of the disease. Thus, such data has led authors to claim that the phenotypical consequences of *Mecp2* deletion are time and region-dependent (Chao et al., 2010). In this regard, dysfunctional GABAergic signaling constitutes a vital element in RTT symptomatology.

The GABAergic signaling system comprises a key pathway commonly disturbed in neurodevelopmental diseases, such as RTT, fragile X syndrome, Dravet Syndrome, neurofibromatosis type I, Tourette syndrome, Down Syndrome and in Autism Spectrum Disorders (Lee and Tierney, 2011; El-Ansary and Al-Ayadhi, 2014; Braat and Kooy, 2015; Kim and Yoon, 2017). GABA is the main inhibitory neurotransmitter in the brain, exerting its actions via the activation of two subtypes of GABA receptors, the ionotropic GABA_A_R and the metabotropic GABA_B_R (Chebib and Johnston, 1999; Rombo et al., 2016). GABA_A_R are ligand-gated receptors responsible for mediating the majority of inhibitory synaptic transmission in the CNS (Reddy, 2010). Structurally, GABA_A_R are heteropentamers with five protein subunits that form the chloride ion channel (Reddy, 2010). GABA_A_R can be assembled by seven different classes of subunits, some of which comprised by different homologous variants (α_1__–__6_, β_1__–__3_, γ_1__–__3_, σ_1__–__3_, δ, θ, ε). Usually, most GABA_A_R are composed by α, β, and γ or δ-subunits (Sieghart, 2006). The binding site for the endogenous modulator GABA is located in the cleft between the α and β subunits. Apart from the GABA binding site, there are several other binding sites in the GABA_A_R which constitute targets for benzodiazepines and barbiturates (Smith, 2002). Furthermore, each pharmacological effect appears to be directly related with a specific binding site on the receptor surface, which depends on the subunit composition of the receptor (Cai et al., 2018). Post-synaptic GABA_A_R are ubiquitously distributed, being responsible for generating the phasic currents in response to presynaptic GABA release. On the other hand, extrasynaptic GABA_A_R are preferentially activated when GABA levels are low, being highly sensitive to extracellular GABA concentrations and responsible for generating non-desensitizing tonic inhibition (Wang, 2011; Cai et al., 2018). Despite being exclusively present in a minority of neurons, several clusters of GABAergic interneurons are responsible for regulating and controlling the majority of excitatory neurons. Thus, if an imbalance in the inhibitory system occurs, the following excessive excitatory output leads to disturbances on the excitation/inhibition balance, ultimately resulting in dysfunction of cognitive processes (Braat and Kooy, 2015). Importantly, genetic alterations in GABAergic signaling system has been shown to be involved in several neurodevelopmental diseases (for a detailed review see Braat and Kooy, 2015).

Evidence regarding dysfunctional GABAergic signaling in RTT has been mainly obtained in studies using RTT-mouse models. Such studies have been gathering substantial evidence confirming the critical involvement of GABAergic transmission in RTT symptomatology, highlighting the clinical relevance of this signaling system. Indeed, an incorrect balance between excitation and inhibition reflecting a dysfunction in GABAergic and glutamatergic signaling systems have been described in *Mecp2*-knockout mice (Dani et al., 2005; Chao et al., 2007; Dani and Nelson, 2009; Calfa et al., 2011). Also, as mentioned before regarding other neuromodulatory systems, the relationship between RTT phenotypical expression and GABAergic signaling is region, time and neuronal population dependent (El-Khoury et al., 2014). Confirming the critical involvement of the GABAergic system in RTT symptomatology, it has been shown that mice with *Mecp2*-deficiency exclusively in GABAergic neurons rapidly develop forepaw stereotyped movements, compulsive grooming, learning and memory deficits, abnormal social behavior, electroencephalography hyperexcitability, lack of motor coordination, severe respiratory dysrhythmias and premature lethality. Additionally, the results of this work also showed that the selective deletion of the *Mecp2* gene in a specific subset of forebrain GABAergic neurons was sufficient to trigger many of the aforementioned symptoms. Thus, these data strongly suggests that the loss of *Mecp2* in GABAergic neurons acts as a pivotal mediator for some characteristic RTT phenotypes (Chao et al., 2010).

The critical relevance of *MECP2* in GABAergic signaling was also shown in other brain regions, such as the hippocampus. Indeed, patch-clamp recordings performed in hippocampal slices obtained from *Mecp2* mutant mice, revealed that hippocampal circuits in CA3 neurons display diminished basal inhibitory rhythmic activity, which, consequently, leaves the circuitry susceptible to hyperexcitability (Zhang et al., 2008). Likewise, in the brain stem, an imbalance between inhibitory and excitatory transmission was reported to occur early at postnatal day 7 in *Mecp2* mutant mice. Additionally, GABAergic transmission was found to be significantly depressed in the same animals, which may be related with deficient presynaptic GABA release and diminished expression levels of subunits α2 and α4 on the post-synaptic GABA_A_R (Medrihan et al., 2008). In the thalamus, *Mecp2* differentially regulates the development of GABAergic synapses in excitatory or inhibitory neurons (Zhang Z.W. et al., 2010). Also, in *Mecp2* mutant mice, electrophysiological deficiencies have been reported in norepinephrinergic neurons of the locus coeruleus which receive GABA_A_ergic inhibitory inputs (Jin et al., 2013a; Zhang X. et al., 2010). In the locus coeruleus, *Mecp2* deficiency leads to simultaneous abnormalities in the pre- and post-synaptic GABAergic component, impairing GABA_A_ergic and GABA_B_ergic post-synaptic inhibitory currents and reducing the presynaptic release of GABA (Jin et al., 2013a). Indeed, it has been described that *Mecp2* deficiency abnormally increases extrasynaptic GABA_A_R activity, an effect suggested to occur as a compensatory response to the deficient GABA-mediate synaptic inhibition (Zhong et al., 2015).

The above-mentioned evidence highlights the critical role of *MECP2* gene for correct GABAergic signaling in different brain regions. Besides brain region and synaptic location of the receptor, in a recent study it was also shown that selective deletion of *Mecp2* from either parvalbumin-positive neurons or somatostatin-positive neurons leads to different phenotypical outcomes, that together comprise almost the full range of RTT-like phenotypes. Specifically, *Mecp2* deletion from parvalbumin-positive neurons leads to motor, sensory, cognitive and social deficits, while ablation of *Mecp2* from somatostatin-positive neurons originated stereotyped behavior and seizures (Ito-Ishida et al., 2015). Recently, reductions in GABAergic transmission in the nucleus tractus solitarius, a key brain region involved in the integration of respiratory sensory information, were reported (Chen et al., 2018).

Although scarce, there is available data collected from human samples supporting findings in rodent models of RTT (Braat and Kooy, 2015). GABA_A_R binding was found to be significantly altered in the basal ganglia (Blue et al., 1999) and in the frontotemporal cortex (Yamashita et al., 1998) in RTT patients. Additionally, a disturbed process of GABAergic neuronal maturation was described in the cerebrospinal fluid of RTT patients (Duarte et al., 2013) (for a more details see Braat and Kooy, 2015). Also, an analysis performed in *post-mortem* human brains, has shown defects in the expression of the GABR gene GABRB3, contained in the human chromosome 15q11-13, in RTT patients (Samaco et al., 2005). Congruently, subsequent evidence obtained with mice models, suggested that *Mecp2* gene is vital for the correct expression of both alleles of GABRB3 in neurons (Hogart et al., 2007).

**FIGURE 1:**
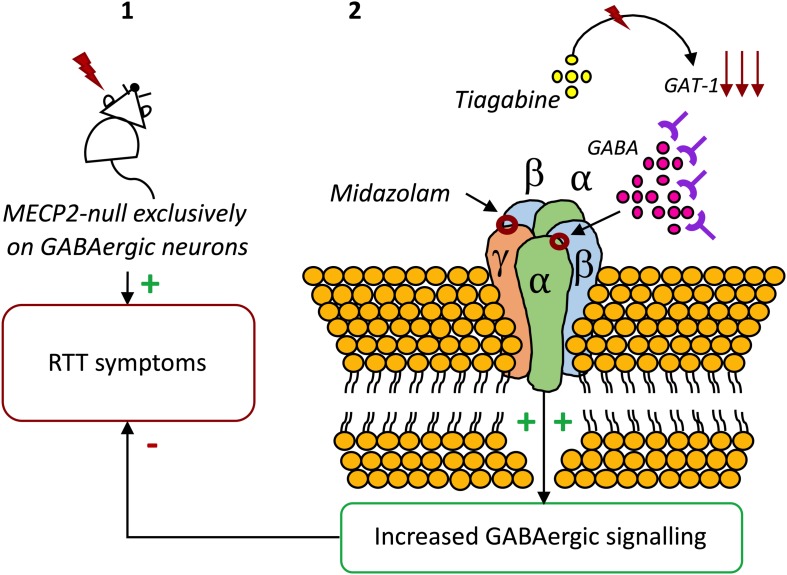
The potential of GABA_A_R modulators on RTT. **(1)** Exclusive deletion of *Mecp2* from GABAergic neurons results in almost the full range of neuropsychiatric symptoms of RTT (Chao et al., 2010). **(2)** Blocking GABA reuptake using tiagabine (El-Khoury et al., 2014) or enhancing GABA_A_R activity with the benzodiazepine Midazolam increases the life-spam and reduces symptoms in *Mecp2* mutant mice (Voituron and Hilaire, 2011).

Considering the anomalies observed in GABAergic signaling in RTT and their crucial role in symptomatology and disease progression, correction of these modifications has been proposed as a therapeutic strategy. Indeed, adjustment of the GABAergic system activity in *Mecp2* mutant mice leads to improvements in several phenotypical features (Braat and Kooy, 2015). In accordance, in a recent study, it was found that an *in vivo* treatment with the inhibitor of GABA reuptake, Tiagabine, significantly increased the life-spam of *Mecp2* knockout mice, although it did not ameliorate motor deficits (El-Khoury et al., 2014). In another study, the benzodiazepine Midazolam transiently abolished breathing abnormalities in *Mecp2* mutant mice (Voituron and Hilaire, 2011). Also, the enhancement of GABA_A_R-mediated signaling, either through blockade of GABA reuptake or through positive allosteric modulation of the GABA_A_R, resulted in a significant reduction on respiratory problems (Abdala et al., 2010). Recently, respiratory dysrhythmia has been related with deficient GABAergic signaling in the Kölliker-Fuse area, or subparabrachial nucleus, a brain area responsible for the regulation of breathing rate. Congruently, boosting GABA transmission reduced respiratory arrhythmia in a RTT-mouse model (Abdala et al., 2016). Also, early exposure to the extrasynaptic GABA_A_R agonist THIP was shown to regulate neuron hyperexcitability on the locus coeruleus, known to be involved in the regulation of breathing (Zhong et al., 2017). Finally, genetic re-expression of *Mecp2* only in GABAergic neurons of male and female *Mecp2* null mice enhanced inhibitory signaling. Also, it extended animal lifespan, mitigated ataxia, apraxia, and social withdrawal, displaying that the restoration of *Mecp2* expression in GABAergic neurons significantly improves the symptomatology of RTT (Ure et al., 2016; Figure 1).

**FIGURE 2:**
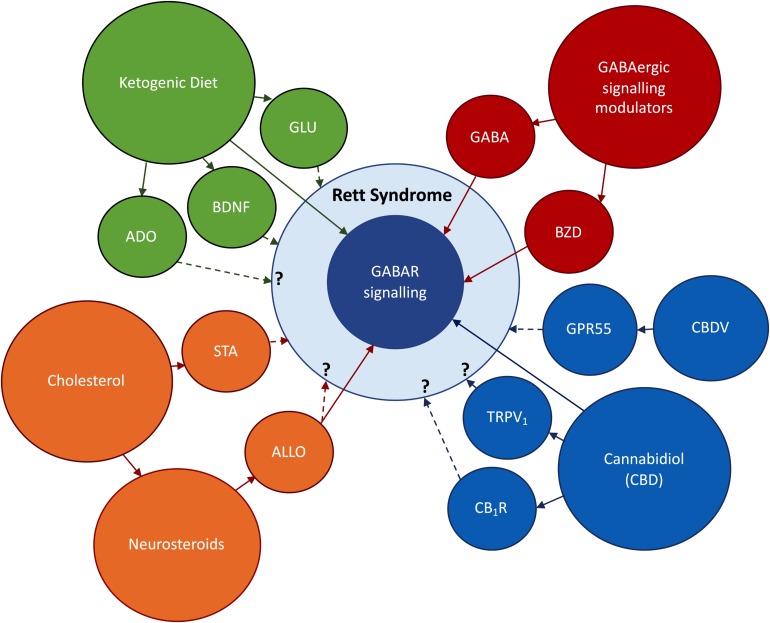
Research topics to be addressed in this review. **In red:** the use of direct and indirect modulators of GABAergic signaling, respectively, benzodiazepines (BZD) and GABA enhancers. **In orange:** Abnormal cholesterol metabolism occurring in RTT, the use of statins (STA) to improve RTT symptoms; the impact of deficits on cholesterol uptake on neurosteroidogenesis; the possible therapeutic actions of the neurosteroid allopregnanolone (ALLO). **In blue:** the use of derivatives of the cannabis plant to ameliorate RTT symptoms; cannabidiol (CBD) has shown promising anti-epileptic effects via GABA-dependent mechanisms; the effects of CBD on CB_1_R and TRPV_1_ and its impact on RTT symptoms are still undisclosed; Cannabidivarin (CBDV) has been identified as a promising therapeutic drug in an RTT-mice model. **In green:** Ketogenic Diet (KD) has potent anticonvulsive actions via GABAergic and glutamatergic (GLU) signaling systems; the KD can also improve RTT symptoms via BDNF-mediated effects or through increases in adenosine production. BNDF signaling is known to be impaired in RTT (Li and Pozzo-Miller, 2014), while unpublished data obtained in our lab points to deregulations in adenosinergic signaling. Direct arrows denote direct interaction with GABAergic signaling system, while doted arrows denote known or undisclosed involvement on RTT pathophysiology.

Taken together, these results emphasize the crucial role of *MECP2* in GABAergic neurons. Also, they demonstrate a clear relationship between modifications in GABAergic signaling and symptomatology in RTT, highlining the potential of drug agents which correct these modifications to improve RTT symptoms. Therefore, on the following sections we will focus our attention on new findings that, directly or indirectly, involve the modulation of GABAergic signaling in RTT (Figure 2).

## RTT, a Brain Disease With a Metabolic Blueprint

### Cholesterol and Neurosteroids in Rett Syndrome

Rett syndrome was initially considered to be a metabolic disease (Justice et al., 2013). Indeed, originally, Dr. Rett, convinced of being in the presence of a disease with a clear metabolic component, named it “cerebroatrophic hyperammonaemia” (Kyle et al., 2018). Hyperammonemia is a clinical condition characterized by increased ammonia levels, which manifests itself in a variety of symptoms and signs, including significant CNS abnormalities (Auron and Brophy, 2012). The fact that hyperammonaemia has been detected only in a minority of RTT patients led to the abandonment of the hypothesis of cerebroatrophic hyperammonaemia as the central cause for RTT (Campos-Castelló et al., 1988; Justice et al., 2013). Recently, however, the metabolic components of RTT and its importance for disease progression have been brought again into light.

Cholesterol is a vital component of the cellular membrane structure and a precursor for numerous signaling molecules (Nagy and Ackerman, 2013). Cholesterol does not cross the BBB and therefore brain cholesterol has to be generated locally, which directly contrasts with other organs that are able of acquiring it from circulating lipoproteins (Pfrieger and Ungerer, 2011). In the brain, the production of cholesterol is a highly compartmentalized process presenting a very delicate balance. Its production takes place in the endoplasmic reticulum and requires, as an energy source, the correct functioning of the mitochondria (Justice et al., 2013). Importantly, mitochondrial function and structure is compromised in both RTT patients and *Mecp2* mutant mouse models (Shulyakova et al., 2017). After synthesis, brain cholesterol must be quickly renewed and turned-over, as it can be rapidly oxidized by reactive oxygen species (ROS), known to accumulate whenever mitochondria dysfunction occurs (Justice et al., 2013). To maintain homeostasis, cholesterol must be converted into the 24S-OHC by the neuron-specific enzyme Cyp46a1 (Lund et al., 2003; Kyle et al., 2018). Thus, a tight regulation of cholesterol homeostasis in the brain assumes particular importance: too much or too little cholesterol is detrimental, negatively impacting on cognitive processes, memory and motor skills (Kyle et al., 2018). Concordantly, a vast number of neurological diseases present abnormal lipid synthesis, storage and recycling (Waterham, 2006), namely, SLOS and Niemann–Pick type C disease, Alzheimer’s, Parkinson’s, and Huntington’s diseases, Amyotrophic lateral sclerosis, Fragile X syndrome, and, as recently discovered, RTT (Justice et al., 2013).

Using *Mecp2* mutant mice, a mutagenesis screening to identify genes that influence RTT-linked phenotypes was performed (Buchovecky et al., 2013; Nagy and Ackerman, 2013). To do so, descendent of *MECP2* mice were screened for limp gasping, tremors and activity, and mice that displayed reduced phenotypic features were bred to establish the hereditability of putative suppressor genes (Buchovecky et al., 2013). This screening highlighted that a mutation in the *Sqle* gene, encoding squalene epoxidase, which is a rate-limiting enzyme in cholesterol biosynthesis, was sufficient to restore function and longevity in *Mecp2* mutant mice. The authors also showed that cholesterol metabolism is perturbed in brains and livers of *Mecp2* mutant male mice, revealing profound and complex dysregulations in cholesterol metabolism. Furthermore, HMG-CoA reductase inhibitors, or statins, were able to ameliorate the systemic imbalance of lipid profile, alleviate motor symptoms and increase longevity in *Mecp2* mutant mice (Buchovecky et al., 2013). Studies with samples collected from human RTT patients have also shown modifications in lipid profile. Directly comparing with age-matched healthy donors, imbalances in both high (HDL) and low (LDL) density lipoprotein levels were found in RTT patients. Accompanying these abnormalities in plasma lipid profile, a marked reduction in SRB1 was detected. SRB1 is ubiquitously expressed, playing vital roles in cellular lipid uptake, mediating, for instance, the uptake of HDL-derived cholesterol in the liver (Sticozzi et al., 2013). Congruently, using freshly isolated human fibroblasts, it has been shown that total cholesterol and LDL levels are significantly increased in RTT patients, while SRB1 expression was quantified has being 70% lower in RTT patients compared with healthy controls (Segatto et al., 2014). On the other hand, and in accordance with data from Buchovecky et al. (2013), cholesterol synthesis was found to be reduced in RTT fibroblasts (Segatto et al., 2014). Therefore, these data provided strong evidence toward the importance of cholesterol homeostasis in RTT. Also, imbalances in cholesterol metabolism can affect the synthesis of steroid hormones, among which neurosteroids can have an important role in several brain diseases, as it will be approached in the following section.

### Neurosteroids: The Way Is Through the GABA_A_R

Neurosteroids were described, for the first time, has having anesthetic and anticonvulsive actions in the late 1940s (Selye, 1941; Selye and Masson, 1942; Clarke, 1973; Reddy and Estes, 2016). Almost half a century later, alphaxolone, a synthetic neurosteroid, was found to increase synaptic inhibition through GABA_A_R activation (Harrison and Simmonds, 1984), which constituted a major advanced in neurosteroid research (Majewska et al., 1986).

**FIGURE 3:**
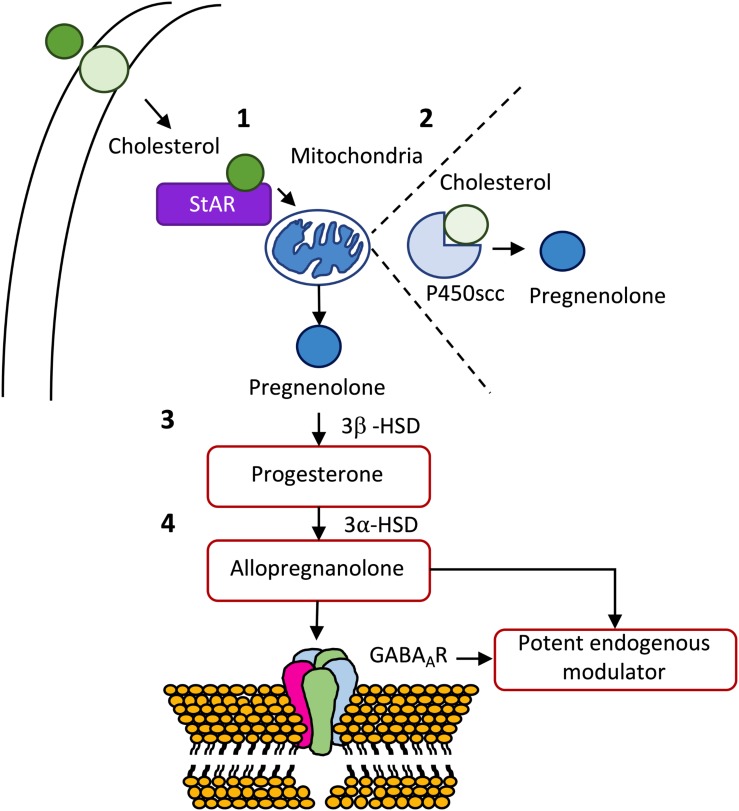
*De novo* synthesis of some important neurosteroids. **(1)** After being transported from the outer to the inner membrane, cholesterol is transported from the outer to the inner membrane of the mitochondria by steroidogenic acute regulatory protein (StAR). **(2)** Once inside the mitochondria, cholesterol is transformed in pregnenolone, the mother of all steroid hormones, by cytochrome P450 cholesterol side-chain cleavage (P450scc) enzyme (CYP11A1). **(3)** Pregnenolone can be, subsequently, transformed into progesterone by 3β-hydroxysteroid dehydrogenase (3β-HSD), which in turn, can originate allopregnanolone through the action of 3α-hydroxysteroid dehydrogenase (3α-HSD). **(4)** Allopregnanolone is viewed as the most potent endogenous modulator of the GABAergic system via interaction with the GABA_A_ receptor (for a detailed review see Melcangi et al., 2014).

Neuroactive steroids are steroid molecules synthesized in the brain that modulate neuronal excitability by rapid non-genomic actions (Reddy, 2010). Cholesterol constitutes the raw material for the biosynthesis of all steroid hormones (Hu et al., 2010). *De novo* synthesis of neurosteroids is a sequential and highly compartmentalized process involving, as first step, the translocation of cholesterol from the cytoplasm to the inner mitochondrial membrane. Once inside the mitochondria, cholesterol is converted into pregnenolone, the precursor of all steroid hormones (Reddy, 2010; Melcangi et al., 2014). Pregnenolone is then transformed into progesterone by the action of the enzyme 3β-HSD (Melcangi et al., 2008). The conversion of cholesterol to pregnenolone in the mitochondria is, consequently, the first rate-limiting step in the biosynthesis of all steroid hormones (Stoffel-Wagner, 2003). ALLO is derived from progesterone by 5α-reductase and 3α-HSD (Liu and Wong-Riley, 2010), and is viewed as the most potent endogenous modulator of the GABAergic system via interaction with the GABA_A_R (Hosie et al., 2006; Reddy, 2010; Figure 3).

Recent studies indicate that neurosteroids have anxiolytic, antidepressant and antipsychotic properties. Furthermore, neurosteroids stimulate neurogenesis, facilitate regeneration of neurons after injury, promote myelinization, and improve cognition (Reddy and Estes, 2016; Cai et al., 2018). The development of new analogous neurosteroid agents with promising therapeutic properties has resulted in four compounds tested in clinical trials for epilepsy, traumatic brain injury, status epilepticus and Fragile X syndrome. Also, several synthetic neurosteroids have been prepared for therapeutic use in the past decades, among which, the best-known are, the aforementioned alphaxolone, and also alphadolone, minaxolone, and ganaxolone (Reddy and Estes, 2016).

Neurosteroids have been implicated in the behavioral profile of some neurologic diseases. In SLOS, an autosomal recessive disorder which develops due to an inborn error in cholesterol metabolism (Tint et al., 1994; Aneja and Tierney, 2008; Lee and Tierney, 2011), impaired neurosteroid synthesis or the synthesis of an inhibitory analog form of neurosteroids on the brain have been proposed to occur (Porter, 2002). Congruently, urinary analysis has allowed the identification of neurosteroid-like compounds in SLOS patients, being foreseeable that this abnormal synthesis also occurs in the brain (Marcos et al., 2004; Lee and Tierney, 2011). In Fragile X syndrome, patients show autism-like phenotypes characterized by cognitive impairment, anxiety, mood swings and behavioral and learning difficulties (Reddy and Estes, 2016). A clinical trial using ganaxolone was found to be safe but produced no significant effects on the outcome measures in the overall population of the study. However, specific subsets of the children with Fragile X syndrome enrolled on the clinical trial, particularly the ones with higher anxiety, lower cognitive abilities and who have frequent seizure episodes, might benefit from treatment with this neurosteroid (Ligsay et al., 2017).

Recently, it has been described that ALLO (Meletti et al., 2017) and progesterone (Meletti et al., 2018) are decreased in cerebrospinal fluid (CSF) of patients affected by status epilepticus. Progesterone levels were found to be 64% lower than in healthy controls (Meletti et al., 2018). Interestingly, neurosteroids are also being proposed for the treatment of schizophrenia, as neurosteroids show promising anti-psychotic potential (Cai et al., 2018).

The therapeutic potential of neurosteroids is mainly related with their ability to rapidly modulate the activity of the GABAergic neurons, known to be involved in the pathophysiology of several psychiatric disorders (Mellédo and Baker, 2002; Dubrovsky, 2005; Marx et al., 2006) and neurodegenerative diseases (Carunchio et al., 2008; Li et al., 2016; Kim and Yoon, 2017; Lozovaya et al., 2018), and commonly disturbed in neurodevelopmental diseases (see section “Dysfunctional GABAR Signalling in RTT: Implications for Symptomatology”). The modulatory effects of neurosteroids on GABA_A_R are unique and extremely complex, depending on several factors, such as, the subunit composition of the receptor, the receptor localization, concentration and the structure of the neurosteroid (Sieghart, 2006; Reddy, 2010; Reddy and Jian, 2010; Wang, 2011; Reddy and Estes, 2016; Cai et al., 2018). Previous studies have shown that specific combinations of subunits in the GABA_A_R confer more or less affinity for neurosteroids, but that ALLO enhances GABA_A_ergic transmission whenever the GABA_A_R are composed by any α subunit (Puia et al., 1993, 2003; Maitra and Reynolds, 1999; Belelli et al., 2002). Importantly, both post-synaptic GABA_A_R and extrasynaptic GABA_A_R are highly sensible to neurosteroid modulation. In particular, extrasynaptic GABA_A_R containing the δ subunit located in specific brain regions as the hypothalamus, hippocampal dentate gyrus and cerebellum, are highly sensitive to neurosteroids, which may open a therapeutic window for the use of neurosteroids in several brain diseases, including RTT (Smith, 2002; Reddy and Estes, 2016). Indeed, neurosteroids have been proposed as a viable alternative to overcome benzodiazepine tolerance, as they can bind and enhance the activity of all GABA_A_R isoforms, including extrasynaptic GABA_A_R containing the highly neurosteroid sensitive δ subunit (Brown et al., 2002; Uusi-Oukari and Korpi, 2010; Meera et al., 2011; Rogawski et al., 2013; Carver and Reddy, 2016).

**FIGURE 4:**
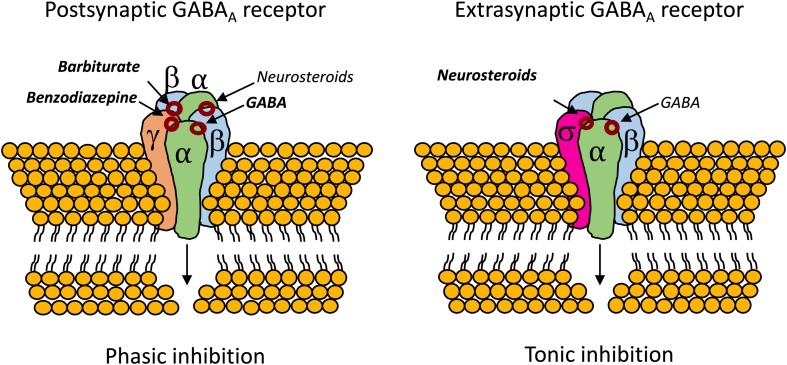
Binding sites for neurosteroids on the postsynaptic and extrasynaptic GABA_A_R (Reddy, 2011; Cai et al., 2018). Contrary to benzodiazepines, which require the mandatory γ subunit, neurosteroids can act on GABA_A_R whenever they are composed by α subunits (Bianchi and Macdonald, 2003). The extrasynaptic GABA_A_R usually contains the highly neurosteroid sensitive δ subunit, which opens an exploitable therapeutic window, especially in cases of benzodiazepine tolerance (Brown et al., 2002; Uusi-Oukari and Korpi, 2010; Meera et al., 2011; Rogawski et al., 2013; Carver and Reddy, 2016).

Also, unlike benzodiazepines, neurosteroids are able of modulating the activity of GABA_A_R that lack the mandatory γ subunit, which confers benzodiazepine sensitivity to the GABA_A_R (Bianchi and Macdonald, 2003). In this regard, neurosteroids have been tested in super refractory status epilepticus with mixed-results (Broomall et al., 2014; Rosenthal et al., 2017; Vaitkevicius et al., 2017). Analogs of ALLO were found to be protective against partial seizures induced by electrical stimulation in animals (Kaminski et al., 2004; Figure 4).

The role of ALLO as a neuroprotective agent starts before birth. Indeed, ALLO levels fluctuate during perinatal periods (Bernardi et al., 1998; Concas et al., 1998; Genazzani et al., 1998; Grobin and Morrow, 2001; Jin et al., 2013b) and its actions are known to contribute to neuroprotection of the fetal brain (Liu and Wong-Riley, 2010). In rats, ALLO levels drop immediately after birth, keeping a steady decline until a temporary elevation at 10 to 14 days postnatal, then decrease to a steady low level 3 weeks after birth (Grobin and Morrow, 2001). The drop in ALLO levels seems to coincide with drastic reductions in progesterone levels immediately after birth (Paoletti et al., 2006). Relevantly, in *MECP2* mutant mice, the reductions in ALLO levels coincide with the onset of RTT symptoms in such animals (Jin et al., 2013b).

**FIGURE 5:**
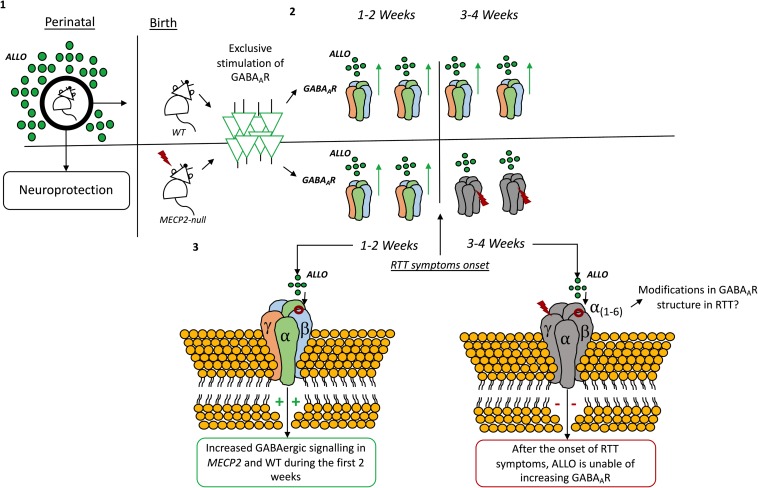
Time-dependent modulation by allopregnanolone on GABA_A_R signaling. **(1)** Allopregnanolone actions are known to contribute to neuroprotection of the fetal brain (Liu and Wong-Riley, 2010). In rats, allopregnanolone levels change immediately after birth and during the first weeks of life (Grobin and Morrow, 2001). **(2)** By exclusively stimulating the GABA_A_-ergic neurons in the locus coeruleus it was found that allopregnanolone increases the amplitude, frequency and decay time of GABA_A_R inhibitory post-synaptic currents (IPSCs) both in wild-type and *Mecp2* mutant animals. **(3)** However, after the first 2 weeks, allopregnanolone effects are lost in *Mecp2*-null animals, coinciding with the onset of RTT symptoms in such animals (Jin et al., 2013b). The drop in allopregnanolone levels seems to coincide with drastic reductions in progesterone levels immediately after birth (Paoletti et al., 2006). Modifications in the composition of the GABA_A_R can also be responsible for the effect (Jin et al., 2013b).

There are very few examples of the use of neurosteroids in RTT available on literature. Jin et al. (2013b), using an optogenetic approach to directly stimulate GABA_A_R in the locus coeruleus, found that ALLO increases the amplitude, frequency and decay time of GABA_A_ergic inhibitory post-synaptic currents (IPSCs). In this study, the authors described a time-dependent modulation of GABA_A_ergic transmission by ALLO in *Mecp2* mutant mice. Like in wild-type mice, during the first 2 weeks of the postnatal period, ALLO increases GABA_A_ergic inhibitory post-synaptic potentials and suppresses neuronal excitability in locus coeruleus neurons of *Mecp2* mutant mice. However, such effect abruptly deteriorates at 3 weeks of age in *Mecp2* deficient mice, which coincides with the onset of RTT symptoms. Therefore, the authors hypothesize that, in *MECP2* mutant mice, declines in progesterone levels after birth may lead to decreases in ALLO biosynthesis which in turn, becomes insufficient to potentiate GABA_A_ergic transmission through the GABA_A_R (Jin et al., 2013b). There is, also, the possibility that the subunit composition of the GABA_A_R shifts during development (Rissman and Mobley, 2011; Datta et al., 2015) or in pathophysiological conditions (Drexel et al., 2013; Govindpani et al., 2017; Kwakowsky et al., 2018) and also that it varies accordingly to temporal and local constrains (Walton et al., 2017). Thus, these modifications in GABA_A_R composition may change the receptor sensibility to neurosteroids in *Mecp2* mutant mice (Jin et al., 2013b) (see Figure 5). Also, GABA_A_R sensitivity toward ALLO can shift due to neurosteroid withdrawal (Gulinello et al., 2001; Mascia et al., 2002; Boggio et al., 2010), long-term steroid administration (Follesa et al., 2002) and age (Shen et al., 2007), amongst other factors [reviewed in Mellon (2007)]. Other studies have shown that ALLO stimulates GABA_A_ergic currents in the hippocampus (Park et al., 2011), cerebellum (Cooper et al., 1999), and neocortex (Puia et al., 2003).

Evidence displaying the existing of a time-dependent modulation of GABA_A_ergic transmission by ALLO in *Mecp2* mutant mice is certainly important, as it shows how the GABAergic system is vulnerable and mutable in RTT. However, there is an ample bulk of evidence displaying that, even in wild-type animals, changes in GABA_A_R sensitivity toward neurosteroids occur during embryonic development and even later in puberty. These changes can potentially lead to different effects of ALLO and other neurosteroids during development (Follesa et al., 2002). Thus, the GABA_A_R subunit composition may shift accordingly to the developmental stage. Therefore, the existing time-dependent GABA_A_R sensitivity to neurosteroids can reflect a changeable requirement of neurosteroid actions by the GABAergic system, which, in turn, may be related with the hormonal milieu of the specific period of life (Colciago and Magnaghi, 2016). Also, either physiological or pathophysiological conditions can change the way neurosteroids act on the GABAergic system. Understanding how the subunit composition of the GABA_A_R shifts in wild-type animals comparing to RTT mouse models, may offer important insight to elucidate the role and therapeutic potential of neurosteroids in RTT. Also, it would be relevant to study how the existent imbalances in cholesterol metabolism can impact the biosynthesis of neurosteroids in RTT.

The effects of ALLO on hippocampal-dependent memory are a great example of how the actions of neurosteroids can shift according to physiological/pathological conditions. GABA_A_R in the dentate gyrus, where ALLO is actively metabolized, display low sensitivity to this neurosteroid. In turn, lower ALLO metabolism in the CA1 region of the hippocampus suggests that local steroid metabolism plays a crucial role in the regulation of GABA_A_R-mediated inhibition in a regionally dependent manner (Belelli and Herd, 2003), specifically in brain regions devoted to memory formation and consolidation (Colciago and Magnaghi, 2016). This is strengthened by observations that ALLO increases neurogenesis, neuronal survival and reduces apoptosis in the hippocampus (Charalampopoulos et al., 2008; Rossetti et al., 2015). ALLO also increases the density of dendritic spines, the number of mature excitatory synapses and induces the formation of clusters of the actin binding protein debrin (Shimizu et al., 2015), which is known to be decreased in the brain of Alzheimer’s disease patients and individuals with mild cognitive impairment (Harigaya et al., 1996; Counts et al., 2012). Congruently, using an Alzheimer’s disease mice model, ALLO restored hippocampal-dependent learning and memory deficits and induced survival of newly generated cells in 3xTgAD mice and non-Tg aged mice (Singh et al., 2012). Thus, in 3xTgAD mice it has been proposed that an early deficit in neurosteroid synthesis may contribute to the cognitive phenotype of AD and that ALLO shows the potential to function as a regenerative therapeutic tool to delay or even prevent characteristic neurogenic and cognitive deficits of Alzheimer’s disease (Wang et al., 2010; Singh et al., 2012). In addition, ALLO was found to significantly reduce amyloid-β accumulation in the hippocampus, cortex and amygdala, to reduce microglia activation and increase expression of liver-X-receptor, pregnane-X-receptor and HMG-CoA-R, three vital proteins involved in cholesterol homeostasis and clearance from the brain (Chen et al., 2011). Despite the clear effects of ALLO on the molecular mechanisms of hippocampal memory, on the other hand, it has been described that ALLO impairs recognition memory consolidation and contextual fear memory in wild-type mice (Misane et al., 2013; Rabinowitz et al., 2014), and spatial learning in rats (Johansson et al., 2002; Matthews et al., 2002; Turkmen et al., 2004). Importantly, *Mecp2* mutant mice display learning and memory impairments, besides the classic breathing abnormalities, hypoactivity, motor deficits, anxiety alterations and stereotypies. Likewise, *Mecp2* mutant mice have reduced hippocampus, amygdala and striatum volumes and an overall reduction in brain volume (Stearns et al., 2007). Therefore, direct research on the possible restorative effects of neurosteroids in RTT could constitute an interesting and potentially successful approach.

### Epilepsy in RTT: CBD to the Rescue?

Epilepsy is fairly a common comorbidity in RTT, with reports showing its presence in 60–80% of patients. In RTT, there is no unambiguous pattern of seizure episodes, and almost all seizure types are reported (Krajnc, 2015). The manifestations of seizures seems to constitute an age-related event (Chapleau et al., 2013), with onset usually in stage II or III (see section “Introduction”), around 4 years of age, but with possibility of maximal onset age between 7 and 12 years (Krajnc, 2015). In RTT, due to limited experience of epilepsy treatment, there are no definitive recommendations regarding treatment approach with the anticonvulsants currently available. Therefore, the choice of treatment for epilepsy in RTT can be challenging (Krajnc, 2015), being considered a major problem in RTT treatment (Chapleau et al., 2013). Additionally, evidence suggests that the prevalence of drug resistance epilepsy in patients with RTT is about the same as in other newly diagnosed epilepsies, in which 20 to 40% of the patients become refractory to treatment (French, 2007). However, prevention and efficient management of epileptic episodes is of major importance in RTT, as the severity and frequency of epileptic episodes are strong determinants to the clinical severity of RTT phenotype (Krajnc, 2015; Clarke and Abdala Sheikh, 2018).

A number of different AED are used in treatment of epilepsy in RTT, either in monotherapy or combined therapy, and its selection varies from case to case (Krajnc, 2015). Regardless of their many mechanisms of action, one of the main targets of several AEDs is the GABAergic signaling system (Greenfield, 2013). For instance, the effects of valproate acid, one of the most commonly prescribed AEDs, on the GABA signaling system (such as, increased GABA turnover and consequent enhancement of synaptic and extrasynaptic inhibition) are important for its antiseizure actions (Löscher, 1989; Greenfield, 2013; Rogawski et al., 2016). On the other hand, the effects of AEDs on GABAergic signaling can be more direct, like the ones of benzodiazepines, which act as direct agonists of GABA_A_R, known to have a specific binding site for this type of drugs. Benzodiazepines are known for augmenting the amplitude (Macdonald and Barker, 1978) or decay time (Tietz et al., 1999) of GABA_A_R-evoked IPSCs (Greenfield, 2013). However, in order to mediate increases in GABA_A_R currents, the receptor must have in its composition a γ subunit and the selectivity of each benzodiazepine depends on the specific α subunit (for detailed review see Riss et al., 2008). The clinical advantages of benzodiazepines relate with its efficacy against many seizure types (Riss et al., 2008), rapidity of onset, minimal toxicity, possibility of administration via several routes and high efficacy track-record (Macdonald and Barker, 1978). However, its use is limited by adverse side effects, such as cognitive impairment and sedation, tolerance, withdrawal and drug interactions (Riss et al., 2008). Tolerance to benzodiazepine treatment has been associated with time-dependent modifications in GABA_A_R subunit composition (Betjemann and Lowenstein, 2015). Namely, the internalization of subunits β_1__–__3_ and γ_2_ on the GABA_A_R, may lead do decreased inhibition of synaptic transmission (Goodkin et al., 2008). Although benzodiazepines are not commonly prescribed as AED in RTT cases, different studies using RTT-mice models have shown the promising effects of benzodiazepines midazolam (Voituron and Hilaire, 2011) and diazepam (Abdala et al., 2010), particularly attenuating respiratory defects.

Recently, natural constituents of the cannabis plant have been emerging as promising AEDs. Importantly, the ECBS stands as a promising pharmacological target in RTT (Vigli et al., 2018; Zamberletti et al., 2019). The ECBS is a complex neuromodulatory system that regulates important physiological processes (Kano et al., 2009), such as, anxiety and stress (Jenniches et al., 2016), social behavior (Wei et al., 2017), motor coordination (El Manira and Kyriakatos, 2010) and memory/learning (Marsicano and Lafenêtre, 2009). Importantly, these are behaviors usually compromised in RTT (Vigli et al., 2018). Cannabinoids modulate neuronal activity mainly via the CB_1_R and CB_2_R. CB_1_R activation is linked with neuroprotection by controlling excitotoxicity via inhibition of excessive excitatory transmission and consequent calcium release (Vendel and de Lange, 2014). Indeed, the control of excitotoxicity constitutes a major physiological role of the ECBS (Kano et al., 2009). Although the CB_1_R is classically denoted as a G_*i/o*_ protein coupled receptor, mainly linked with inhibitory effects, CB_1_R coupling to G proteins is mutable. CB_1_R is now believed to have very few “intrinsic” properties, being the cellular consequences of its activation likely a reflex of cell type and/or location in which the receptor is situated, their activity in different brain regions and due to temporal constrains (Busquets-Garcia et al., 2018). Contrary to initial belief, which placed CB_2_R exclusively in the immune system (Kano et al., 2009), CB_2_R are now known to be present in neurons, microglia and astrocytes (for a review see Solymosi and Kofalvi, 2017). CB_2_R are involved in the control of glial activation and inflammation and on the regulation of neuronal excitability (Solymosi and Kofalvi, 2017). Moreover, CB_2_R levels can increase in neurons and astrocytes under very specific circumstances, such as, in situations of neuroinflammation and in certain diseases (Onaivi et al., 2012; Di Marzo et al., 2015; Fernández-Ruiz et al., 2015). Related with its physiological roles, the lack of psychoactivity associated with CB_2_R activation, has made this receptor a particular promising therapeutic target for cannabis-based therapies (Cassano et al., 2017). For thorough reviews on the ECBS and its actions, natural cannabinoids and their molecular targets, and the clinical applications of cannabinoids (see Kano et al., 2009; Morales et al., 2017; Solymosi and Kofalvi, 2017).

Today, more than 120 phytocannabinoids (natural occurring cannabinoids) have been identified as constituents of the cannabis plant. The most abundant cannabinoids in the cannabis plant are delta-9-tetrahydrocannabinoid, or Δ^9^-THC, followed by Δ^8^-THC, CBN, CBD, CBG, CBC, THCV, CBV, and CBDV (Morales et al., 2017). From all the natural constituents of the cannabis plant, CBD, a non-psychoactive cannabinoid (Loewe, 1946; Pertwee, 2009), is being widely studied given its high therapeutic value. CBD has been shown, both in humans and rodents, to have antiseizure (Jones et al., 2010; Pamplona and Coan, 2017; Perucca, 2017; Zaheer et al., 2018), anti-inflammatory (Malfait et al., 2000; Nagarkatti et al., 2009; Petrosino et al., 2018), antioxidant (Hampson et al., 2000), and anti-psychotic properties (Zuardi et al., 1991, 2012; Iseger and Bossong, 2015), to have neuroprotective effects (Iuvone et al., 2009; Fernández-Ruiz et al., 2013), to reduce nausea (Parker et al., 2011; Mersiades et al., 2018) and to work as an anxiolytic and anti-depressive drug (de Mello Schier et al., 2014; Blessing et al., 2015; Zuardi et al., 2017). Furthermore, CBD is known to potentiate the clinical efficacy of Δ^9^-THC, increasing the durability of its beneficial effects, while preventing its psychoactive effects (Russo and Guy, 2006; Solymosi and Kofalvi, 2017). CBD acts as a surprisingly high potent antagonist at both the CB_1_R and the CB_2_R (Thomas et al., 2009). Recently, it has also been proposed that CBD acts as a negative allosteric modulator (NAM) at the CB_1_R (Laprairie et al., 2015). Many of CBD therapeutic properties have been suggested to occur due to its effect at the TRPV_1_ (Morales et al., 2017).

The medical use of cannabis has been increasing and the full potential of the cannabis plant is yet to be unfold. Cannabis-based drugs are been used, or proposed for use, in neuropathic pain and muscle spasticity associated with Multiple Sclerosis (Fitzpatrick and Downer, 2017; Solymosi and Kofalvi, 2017; Rice and Cameron, 2018), neurodegenerative diseases (Fagan and Campbell, 2014; Basavarajappa et al., 2017; Navarro et al., 2018), chronic pain (Carter et al., 2015; Pascual et al., 2018), to alleviate AIDS-related weight loss (Badowski and Perez, 2016; Rock and Parker, 2016) and to reduce vomiting and nausea associated with chemotherapy (Badowski and Yanful, 2018). Most relevantly for this work, cannabinoid use is being increasingly proposed as a very promising anti-epileptic drug, even in cases of treatment-resistant epilepsy (Maa and Figi, 2014; O’Connell et al., 2017; Pamplona and Coan, 2017). In a recent clinical trial, CBD significantly reduced convulsive-seizure frequency in Dravet syndrome patients (Devinsky et al., 2017). In a double-blind placebo-controlled trial, 120 children suffering with Dravet syndrome and drug-resistance epilepsy received either CBD (oral solution 20 mg/kg per day) or placebo over 14 weeks. In the CBD group, 43% of the patients had a reduction of 50% in seizure frequency, as opposed to a 27% decrease in the placebo group. Furthermore, 5% of the patients treated with CBD became seizure-free compared with a 0% incident in the placebo group. On the CGIC, a Likert-like scale questionnaire which allows assessing improvement after a treatment comparing with a baseline period (ranging from 1, very much improved to 7 very much worse), in the CBD group, 62% of the patients increased at least one category on the CGIC compared with 27% on the placebo group. Treatment with CBD provoked more adverse side effects reflected on diarrhea, vomiting, fatigue, pyrexia, somnolence, and some abnormalities on liver-function tests (Devinsky et al., 2017). About 1 year after this clinical trial, the FDA approved Epidiolex^®^ (CBD) oral solution for treatment in Dravet and Lennox–Gastaut syndromes, two diseases that, in resemblance with RTT, present refractory epilepsy and high mortality rates. Also, Nabiximols (Sativex^®^), a combination of CBD and Δ^9^-THC, is approved to treat muscle spasticity in Multiple Sclerosis (Novotna et al., 2011), an abnormality in muscle tone present in RTT (Kyle et al., 2018).

The mechanism by which CBD exerts anticonvulsive effects is still not completely clarified. Recently, however, using human recombinant GABA_A_R, CBD was shown to act as a positive allosteric modulator of the GABA_A_R, with the effects being selective for the β-subunit (higher in receptors with β2/β3 over β1 subunits) and the maximum effect being detected in receptors which included the α2-subunit (Bakas et al., 2017). The authors hypothesized that these results may explain the anticonvulsant and anxiolytic properties of this compound (Bakas et al., 2017). Indeed, previously, it was reported that CBD attenuated seizures in two well characterized seizure models, the acute pilocarpine TLE and the penicillin model of partial seizure (Jones et al., 2012). Curiously, in the acute epilepsy model using pilocarpine, a muscarinic acetylcholine receptor agonist, the antiseizure effects of CBD were modest and the drug was not able of reducing mortality and severity of episodes. On the other hand, using the penicillin model, a selective antagonist of the GABA_A_R, CBD had a powerful antiseizure effect, significantly reducing the number of animals experiencing tonic-clonic episodes and their mortality. Importantly, CBD exerted only minor negligible motor effects (Jones et al., 2012). At the time, the authors speculated that the antiseizure effect of CBD could be occurring via a GABAergic-mediated mechanism (Jones et al., 2012). In support of this hypothesis, a previous study had shown an anticonvulsive action of CBD on several seizure models mediated *via* disinhibition of GABA release (Consroe et al., 1982; Jones et al., 2012). In a very recent study, CBD was shown to have antiseizure effects in several models of acute epilepsy (Patra et al., 2019). Additionally, this study showed, for the first time, that chronic CBD administration improves seizure burden ratio, attenuates cognitive impairment and reduces motor comorbidities, well after the onset of symptoms in the RISE-SRS TLE (Patra et al., 2019). Thus, the results of this study are particularly relevant for research in RTT, considering the positive effects of a chronic administration of CBD on both seizures and motor impairment, which constitute two common comorbidities in RTT. Additionally, cognitive impairment is also characteristic of RTT and, therefore, finding that CBD improves cognition in models of acute and chronic epilepsy in also relevant for cannabinoid-related research in RTT.

Direct evidence of cannabinoid use in RTT mice models is objectively scarce in literature. To the best of our knowledge, there are only two reports evaluating the impact of a chronic treatment with a phytocannabinoid in an RTT-mice model. In both these reports (Vigli et al., 2018; Zamberletti et al., 2019), the authors used CBDV, a propyl analog of CBD devoid of psychoactive actions (Morales et al., 2017). CBDV has very weak activity at the CB_1_R and CB_2_R (Hill et al., 2013; Rosenthaler et al., 2014; Morales et al., 2017) and it has been proposed to activate the TRPV1 (Iannotti et al., 2014). Additionally, an effect of CBDV on the deorphanized cannabinoid receptor GPR55 has been described. Besides some cannabinoids, GPR55 is also activated by LPI. Notably, it has been found that CBDV is a potent inhibitor of LPI-induced GPR55 signaling (Lauckner et al., 2008; Anavi-Goffer et al., 2012). As stated in Vigli et al. (2018), GPR55 has been implied in important cognitive processes, such as, spatial memory regulation (Marichal-Cancino et al., 2018), social behavior (Kramar et al., 2017) and motor function (Bjursell et al., 2016). Moreover, in a recent work, CBDV was shown to attenuate seizures and social withdrawal in a mice model of Dravet syndrome (Kaplan et al., 2017). Concerning cannabinoids and RTT, the administration of CBDV by intraperitoneal injection (escalating dose ranging from 2 to 100 mg/kg of body weight) to *Mecp2*-308 male mice during 14 days, rescued sociability impairments, improved the general health status and increased the brain weight of these animals. Furthermore, a molecular analysis of GPR55 levels, revealed an up-regulation of this receptor in the hippocampus (Vigli et al., 2018). These results showed, for the first time, the clinical potential of non-psychoactive constituents of the cannabis plant and also the important role of the GPR55 as a promising drug-target on RTT. Very recently, a new study using CBDV has confirmed the potential of this particular phytocannabinoid in RTT. In this study, chronic administration of CBDV (0.2 to 2 mg/kg) was shown to completely rescue cognitive deficits, delaying neurological (although transiently) and motor impairments in male *MECP2* mutant mice (Zamberletti et al., 2019). Importantly, CBDV administration normalized BDNF and insulin growth factor 1 (IGF1) levels and restored normal signaling in the PI3K/AKT/mTOR pathway at an advanced stage of the disease. Interestingly, *Mecp2* deletion in this RTT mouse model provoked upregulation of CB_1_R and CB_2_R and CBDV treatment led to a normalization of these changes (Zamberletti et al., 2019). Although these two independent studies suggest a promising role of CBDV as a therapeutic drug in RTT, the fact that different molecular modifications in the ECBS were detected in each model, highlight the necessity of further studies.

The promising antiseizure effects of CBD, even in cases of refractory-epilepsy, observed in both clinical trials with humans and in laboratory animals, the effects of combinations of CBD and Δ^9^-THC in controlling muscle spasticity and motor symptoms, and the positive results of CBDV administration in two different mouse models of RTT, place cannabinoids as a viable therapeutic strategy in RTT. Moreover, CBD positively modifies impairments in motor, cognitive and social processes in animal models, further highlighting the potential of cannabinoid molecules to tackle RTT-symptomology.

### The Use of the Ketogenic Diet

Around 100 years ago, it was noted that a high fat diet could have long-lasting effects in preventing seizures without inducing significant caloric deprivation (Rogawski et al., 2016). These findings resulted on the creation of the KD, trademarked by an increase in the production in the liver of ketones bodies, such as BHB, acetoacetate and acetone (Peterman, 1924; Freeman and Kossoff, 2010; Rogawski et al., 2016). The fact that the KD was created in a time where the available AEDs were very limited, contributed for a rapid gain in popularity of this diet (Rogawski et al., 2016). However, the development of the antiepileptic drug phenytoin substantially decreased the use of the KD. In the last 30 years, interest in using the KD resurged, especially in children presenting refractory epilepsy (Rogawski et al., 2016).

An abnormal increase of ketone bodies in the blood was early suggested as the underlaying mechanism of the anticonvulsive properties of low carbohydrate KD (Talbot et al., 1927; Huttenlocher, 1976). Subsequently, many other possible mechanisms were enounced, such as, pH changes, anticonvulsive effects of hyperlipidemia, effects of the KD directly on sodium and potassium balance, and also an effect on cerebral metabolism, reflected on the change of glucose, as the main source of energy, to BHB, as a consequence of carbohydrate restriction (for details see Huttenlocher, 1976). To clarify these questions, Peter Huttenlocher designed a new KD in which ketonemia was induced by feeding of MCT. In his report of 1976, the MCT diet was administered to 18 children and he observed no differences in hyperlipidemia, no significant changes in pH, considerable reductions in blood glucose levels in one-third of the children and a gradual increase in plasma BHB and acetoacetate levels during a diet that was kept from 3 months to 4 years. Furthermore, plasma levels of BHB showed a significant correlation with the anticonvulsive effects of the MCT diet and, along with ketonemia, were rapidly abolished by intravenous infusion of glucose (Huttenlocher, 1976). Today, the mechanisms behind the anticonvulsive actions of the KD are fairly well characterized (for a detailed review see Rogawski et al., 2016).

One of the proposed mechanisms of action of the KD are increases in the synthesis of GABA and decreases in the synthesis of excitatory neurotransmitters, such as glutamate (Rogawski et al., 2016). Indeed, it has been suggested that one of the anticonvulsive mechanisms of the KD relates with modifications in the way glutamate is processed in the brain. Particularly, due to a more active astrocytic metabolism, the conversion of glutamate to glutamine is increased, resulting in a more efficient removal of glutamate and increased GABA synthesis (Yudkoff et al., 2008). A study with 26 children suffering from refractory epilepsy, revealed that the KD increases levels of GABA in the cerebrospinal fluid, without affecting glutamate concentration. In this study, the results showed higher GABA levels in the responders to the diet compared with non-responders. Furthermore, the anticonvulsive action of the diet was more evident (90% reduction in seizures) in children who had higher levels of GABA at the beginning of the treatment and in whom a gradual increase in GABA levels during treatment was registered. Also, the anticonvulsive effects of the diet seem to be age-dependent, with younger children being the best responders (Dahlin et al., 2005). However, studies with animal models performed to confirm a GABAergic-mediated mechanism of anticonvulsive actions of the KD have produced mixed-results (Hartman et al., 2007). If, on one hand, the KD administered to mice fails to confer protection against the clonic seizures induced by GABA_A_R antagonist pentylenetetrazol, on the other hand, it is much more effective protecting against seizures induced by the maximal electroshock test, a test in which the classical anticonvulsive drugs targeting the GABAergic system only confer weak protection (Uhlemann and Neims, 1972; Hartman et al., 2007). On the contrary, the KD was found to protect against seizures induced by GABA_A_R antagonists pentylenetetrazol, bicuculline, and picrotoxin in rats (for a detailed review see Hartman et al., 2007). It has also been suggested that ketone bodies, such as acetoacetate, compete with chloride for the allosteric binding site at the vesicular glutamate transporter, thus inhibiting exocytotic glutamate release (Juge et al., 2010). Additionally, the authors of this work showed that acetoacetate protected against seizure-like activity induced by 4-aminopyridine (Juge et al., 2010). Recently, using the spontaneously epileptic Kcna1-null mice, the ketone body BHB was shown to exert anti-seizure effects, restore impairments in synaptic plasticity processes and raise the threshold of mitochondrial permeability transition (Kim et al., 2015; Rogawski et al., 2016).

Concerning the clinical use of the KD in RTT, Haas et al. (1986), reported a clinical study were the MCT KD was used in 7 girls diagnosed with RTT who presented anticonvulsive resistant seizures. Among the 5 girls capable of tolerating the diet, the KD improved seizure control, slightly ameliorated behavior and motor skills and increased bodyweight. The results from this work led the authors to suggest that, considering the defects in the carbohydrate metabolism occurring in RTT, the KD stands as a logical choice in this disease (Haas et al., 1986). Surprisingly, until very recently, this was the only clinical report of the use of KD in RTT. Indeed, evidence of the application of the KD in RTT is extremely hard to find in the literature. In Liebhaber et al. (2003) reported a case of an RTT patient who was treated with the KD for 4 years. The treatment started when the patient was 8 years old and presented a refractory epilepsy. The diet led to a 70% reduction in seizure frequency and improved contact and behavior. In Giampietro et al. (2006) reported a case with a female patient suffering from an atypical RTT variant in which the KD significantly reduced seizure occurrence. In 2011, a long-term follow-up of the use of the KD in 226 patients with refractory epilepsy was reported. This report included one patient with RTT, which presented a 75–99% seizure reduction after KD (Caraballo et al., 2011). Evidence on the use of the KD in RTT-mouse models is also scarce. In the only direct approach available in the literature, it has been described that a restricted KD positively impacts anxiety and motor measures in a male mutant *Mecp2* mice. However, the authors also showed that the results of the KD were very similar to the ones from a calorie restriction diet, concluding that, most likely, it was the caloric restriction that produced the positive outcomes, rather than the composition of the KD by itself (Mantis et al., 2009).

Reports on the use of the KD in autism spectrum disorders and other brain diseases are also available in literature. With relevance for the phenotypic expression of RTT, it has been described that 4 weeks of a KD regime increases the social behavior of wild-type rats, registered in three different social interaction protocols. On the other hand, the exogenous administration of ketone bodies had no effects on social interaction in the same paradigms (Kasprowska-Liśkiewicz et al., 2017). Also, in an EL mice model (Meidenbauer et al., 2011), which presents autism spectrum disorders phenotype and comorbid epilepsy, the KD was shown to improve sociability and reduce repetitive behavior (Ruskin et al., 2017). Using the same animal model, the KD was shown to induce a 1 month delay in epileptogenesis (Todorova et al., 2000) and to have anticonvulsive actions (Mantis et al., 2004). In an Huntington’s disease mice model, the KD was shown to produce positive outcomes on weight lose without affecting cognitive processes (Ruskin et al., 2011). More recently, in an animal model of autism induced by prenatal exposure to valproic acid, the KD was, once again, associated with improvement in social behavior (Castro et al., 2017).

Another potential mechanism of action underlying the anticonvulsive effects of the KD involves the modulation of adenosine receptor activity (Masino et al., 2013; Rogawski et al., 2016). Adenosine is an ubiquitous neuromodulator affecting the action and activity of several neurotransmitter receptors (Sebastião and Ribeiro, 2015). A_1_R activity mediates the inhibitory effects of adenosine, including inhibition of neurotransmitter release and changes in post-synaptic membrane conductance (Dunwiddie and Masino, 2001). On the other hand, the A_2A_R mediates the excitatory effects of adenosine by coupling to G_*s*_ proteins, consequently leading to stimulation of adenylate cyclase, increases in intracellular cAMP levels and PKA activation (Sebastião and Ribeiro, 2015). Relevantly, caffeine, the most widely consumed legal drug in the world, is a non-selective antagonist at the A_1_R and A_2A_R (Fredholm et al., 1999). Recently, the KD was demonstrated to suppress seizures in a transgenic mice model with spontaneous seizures by activation of the A_1_R (Masino et al., 2011) and similar results were shown in an *in vitro* mimic of the KD (Kawamura et al., 2010). Adenosine is directly synthetized from ATP and given that ATP levels are increased in the KD it is feasible that it results in an increase in adenosine synthesis (Rogawski et al., 2016). Adenosine receptor activity, particularly A_2A_R activity, is also known to directly facilitate the actions of the important BDNF (Diógenes et al., 2004, 2007; Fernandes et al., 2008; Fontinha et al., 2008; Vaz et al., 2015). Importantly, BDNF expression is impaired in RTT and it has been suggested that it can have important therapeutic actions in RTT (reviewed in Katz, 2014; Li and Pozzo-Miller, 2014).

In conclusion, if, on one hand, it is particularly well accepted and proven that the KD has powerful antiseizure actions (although the mechanisms of action are still not completely described) (Vining et al., 1998; Hassan et al., 1999; Neal et al., 2008), on the other hand, reports on the application of this diet to RTT cases and in RTT-mice models are scarce. The lack of research is even more surprising considering the positive results shown in both clinical studies with humans and in RTT mouse-models. Relevantly, a prospective study with 145 children aged between 2 and 16 years, suffering from intractable epilepsy and who had never experienced the KD, showed that 3 months of diet resulted in a 75% seizure reduction rate, with 38% of the children in the experimental group experiencing a reduction of 50% in seizures. Also, five children experienced reductions of 90% in seizures and one child became seizure free (Neal et al., 2008). Importantly, in children allocated to the control group who continued their usual treatment with AED, some of them experienced worsening of symptoms and none became seizure-free (Neal et al., 2008; Masino et al., 2013).

## Conclusion and Final Remarks

The present review summarizes important recent evidence concerning metabolic, synaptic, functional and molecular dysfunctions occurring in RTT (see Table 1). Due to the ubiquitous role of *MECP2* gene, it has been a tremendous challenge to comprehend and characterize the mechanisms through which *MECP2* mutations lead to symptomatology in RTT. With the development and improvement of gene-editing technologies, research in RTT has drifted from “therapy-directed” to “cure-oriented” investigation. However, the path for a definitive cure is paved with many challenges and, most likely, this achievement in not around the corner. Meanwhile, it is paramount to better understand this disease and to improve the available treatment by exploring new therapeutic avenues. In this work, we reviewed promising evidence on different research lines that may have an important impact in the field. From modulators of GABAergic signaling, to cannabinoids and the KD, and cleverly exploiting the metabolic features of this disease, an ample bulk of evidence has been gathered, creating a plethora of research lines to be followed in the future. RTT is a devastating disease, both for the patient and for the caregivers, and clinical and preclinical research directed at finding new therapeutic approaches are more important than ever, now that we are beginning to understand some of the mechanisms of this disease.

**TABLE 1:** Summary of the main topics of this review and future directions.

**From cannabinoids and neurosteroids to statins and the ketogenic diet: new therapeutic avenues in Rett syndrome?**				
***GABAergic signaling***	***Cholesterol metabolism***	***Neurosteroids***	***Cannabinoids***	***Ketogenic diet***
The GABAergic signaling system comprises a key pathway commonlydisturbed in neurodevelopmental diseases (Braat and Kooy, 2015).	Cholesterol metabolism is abnormal in brain and livers of *Mecp2* mutant mice (Buchovecky et al., 2013).	Allopregnanolone (ALLO) is the most powerful endogenous allosteric modulator of the GABA_A_R (Hosie et al., 2006; Reddy, 2010).	The endocannabinoid system (ECBS) is involved in the regulation of several behavioral processes found to be compromised in RTT (Vigli et al., 2018).	The ketogenic diet (KD) has strong antiepileptic actions (Rogawski et al., 2016). The anticonvulsive actions of the KD can be mediated via GABAergic signaling mechanism (Rogawski et al., 2016), or be related with increases in adenosine and BDNF signaling (Masino et al., 2011).
In RTT, GABA_A_ergic transmission is intimately related with symptoms and disease progression (Chao et al., 2010; El-Khoury et al., 2014).	A mutation in the Sqle gene was sufficient to restore function and longevity in *Mecp2* mutant mice (Buchovecky et al., 2013).	Neurosteroids are being proposed as an alternative to benzodiazepines (Reddy and Estes, 2016) and also as antipsychotics (Cai et al., 2018).	Cannabis-based therapies are being proposed as anti-epileptic drugs (AED) even in cases of drug resistant epilepsy (Maa and Figi, 2014; O’Connell et al., 2017; Pamplona and Coan, 2017).	The use of the KD in RTT has shown promising results in controlling epilepsy (Haas et al., 1986; Liebhaber et al., 2003; Giampietro et al., 2006; Caraballo et al., 2011).
An incorrect balance between excitation and inhibition reflecting a dysfunction in GABAergic and glutamatergic signaling systems have been described in *Mecp2*-KO mice (Chao et al., 2007; Calfa et al., 2011).	Statins ameliorate the systemic imbalance of lipid profile, alleviated motor symptoms and conferred increased longevity in Mecp2 mutant mice (Buchovecky et al., 2013).	In *MECP2* mutant mice, allopregnanolone increases the amplitude, frequency and decay time of GABA_A_R IPSCs (Jin et al., 2013b).	Nabiximols (Sativex), a combination of CBD and Δ^9^-THC, is approved to treat muscle spasticity in Multiple Sclerosis (Novotna et al., 2011), a comorbidity frequently reported in RTT (Kyle et al., 2018).	The KD has also shown positive results in refractory epilepsy and in intractable epilepsy (Neal et al., 2008; Rogawski et al., 2016).
The relationship between RTT phenotypical expression and GABAergic signaling is region, time and neuronal population dependent (Chao et al., 2010; El-Khoury et al., 2014).	In samples collected from RTT patients the total cholesterol level is altered (Sticozzi et al., 2013; Segatto et al., 2014).	Changes in subunit composition on the GABA_A_R might be responsible for different responses to allopregnanolone (Paoletti et al., 2006; Jin et al., 2013b).	Epidiolex (CBD) has been approved to treat epilepsy in Dravet syndrome (Devinsky et al., 2017), a disease that present refractory epilepsy and high mortality rates. The antiepileptic actions of CBD can be mediated via GABAergic signaling (Bakas et al., 2017).	In *Mecp2* null mice models, the KD ameliorated anxiety and motor measurements, although clarification is required (Mantis et al., 2009).
*MECP2* absence exclusively in GABAergic neurons has been found to trigger almost the full range of RTT symptomatology in mice (Chao et al., 2010).	Modifications in mitochondrial structure and function have been described (Shulyakova et al., 2017). Cholesterol influences the production of neurosteroids (Reddy, 2010).	Reductions in neurosteroids were found on status epilepticus (Meletti et al., 2017, 2018).	Administration of CBDV to *Mecp2*-null mice rescued sociability impairments, improved the general health status and increased the brain weight of these animals (Vigli et al., 2018).	In Autism and Huntington’s disease mice models, the KD has been shown to improve sociability, to reduce repetitive behavior and increase body weight (Todorova et al., 2000; Mantis et al., 2004; Meidenbauer et al., 2011; Ruskin et al., 2011, 2017; Castro et al., 2017; Kasprowska-Liśkiewicz et al., 2017).
The use of benzodiazepines, acting as agonists of the GABA_A_R, improve RTT symptoms in mice (Voituron and Hilaire, 2011).		In SLOS, an abnormal synthesis of neurosteroids occurs (Marcos et al., 2004; Lee and Tierney, 2011). Ganaxolone showed promising results in a clinical trial in Fragile X syndrome (Ligsay et al., 2017).		
Íncreasing GABA availability by blocking its reuptake with Tiagabine, has been shown to reduce RTT symptoms.				

**Future directions**				
To better characterize the modifications occurring in GABA_A_R composition during RTT progression (Jin et al., 2013b).	To comprehend the cellular mechanisms behind the dysregulations in cholesterol metabolism (Buchovecky et al., 2013).	To directly evaluate the impact of neurosteroid treatment in *Mecp2* mutant mice phenotype.	Study the ECBS in RTT mice models.	Reinstate the interest in the use of the KD in RTT, particularly considering its antiseizure actions, advancing with new studies, both in humans and in RTT-mice models.
To further evaluate the impact of strategies which, directly or indirectly, increase GABAergic signaling in RTT mice models.		To understand how modifications occurring in GABA_A_R composition during RTT progression affect neurosteroid actions (Jin et al., 2013b).	Understand the impact of CBD and THC in RTT symptoms in trials with humans and in preclinical studies using RTT mice models.	

Summarizing, in the future it would be interesting to: (1) better characterize the modifications occurring in GABA_A_R composition during RTT disease progression; (2) study how cholesterol imbalances impact on neurosteroid production in RTT and their potential therapeutic use in this disease; (3) disclose the cellular mechanisms behind the dysregulations in cholesterol metabolism; (4) directly study the immense potentiality of CBD and other cannabinoids on RTT mouse-models and in controlled clinical trials with humans; (5) reinstate the interest in the use of the KD in RTT, particularly considering its antiseizure actions, advancing with new studies, both in humans and in RTT-mouse models; (6) to design further studies on the therapeutic properties of statins in RTT, and (7) continue to research mitochondria dynamics in RTT and its role on the disease progression and symptomatology.

## Author Contributions

FM and MD wrote the manuscript. CM-L revised and assisted on manuscript writing. AS revised the manuscript. All authors contributed to the final version of the manuscript.

## Conflict of Interest Statement

The authors declare that the research was conducted in the absence of any commercial or financial relationships that could be construed as a potential conflict of interest.

## Abbreviations

24S-OHC: oxysterol 24(S)-hydroxycholesterol
3xTg-AD: triple-transgenic mouse model of AD
3 α-HSD: 3 α-hydroxysteroid dehydrogenase
3 β-HSD: 3 β-hydroxysteroid dehydrogenase
A_1_R: adenosine A_1_ receptor
A_2A_R: adenosine A_2A_ receptor
AED: anti-epileptic drugs
ALLO: allopregnanolone
ATP: adenosine triphosphate
BBB: blood brain barrier
BDNF: brain-derived neurotrophic factor
BHB: β-hydroxybutyrate
BZD: benzodiazepines
CA1: cornu ammonis 1
cAMP: cyclic adenosine monophosphate
CB_1_R: cannabinoid receptor 1
CB_2_R: cannabinoid receptor 2
CBC: cannabichromene
CBD: cannabidiol
CBDV: cannabidivarin
CBG: cannabigerol
CBN: cannabinol
CBV: cannabivarin
CDKL5: cyclin-dependent kinase-like 5
CGIC: seven-category caregiver global impression of change questionnaire
CNS: central nervous system
CYP11A1: cytochrome P450 cholesterol side-chain cleavage (P450scc) enzyme
Cyp46a1: cholesterol 24-hydroxylase
ECBS: endocannabinoid system
FDA: United States Food and Drug Administration
FOXG1: forkhead box protein G1
GABA: gamma-aminobutyric acid
GABA_A_R: GABA_A_ receptor
GABA_B_R: GABA_B_ receptor
GABR: GABA_A_ receptor subunit
GLU: glutamate
GPR55: G-protein coupled receptor 55
HDL: high density lipoprotein
HMG-CoA: 3-hydroxy-3-methylglutaryl-coA
HMG-CoA-R: 3-hydroxy-3-methylglutaryl-coA reductase
IPSCs: post-synaptic inhibitory potentials
KD: ketogenic diet
LDL: low density lipoprotein
LPI: l- α-lysophosphatidylinositol
MCT: medium chain triglycerides
*MECP2*: methyl-CpG-binding protein 2
PKA: protein kinase A
RISE-SRS: reduced intensity status epilepticus–spontaneous recurrent seizures
RTT: Rett syndrome
SLOS: Smith–Lemli–Opitz syndrome
Sqle: squalene monooxygenase
SRB1: scavenger protein B1
STA: statins
StAR: steroidogenic acute regulatory protein
Tg: transgenic
THCV: Δ ^9^-tetrahydrocannabivarin
THIP: GABA_A_R agonist gaboxadol
TLE: model of temporal lobe epilepsy
TRPV_1_: transient receptor potential cation channel subfamily V member 1
Δ ^8^-THC: Δ ^8^-tetrahydrocannabinol
Δ ^9^-THC: Δ ^9^-tetrahydrocannabino.
